# Low Incidence of Spontaneous Type 1 Diabetes in Non-Obese Diabetic Mice Raised on Gluten-Free Diets Is Associated with Changes in the Intestinal Microbiome

**DOI:** 10.1371/journal.pone.0078687

**Published:** 2013-11-13

**Authors:** Eric V. Marietta, Andres M. Gomez, Carl Yeoman, Ashenafi Y. Tilahun, Chad R. Clark, David H. Luckey, Joseph A. Murray, Bryan A. White, Yogish C. Kudva, Govindarajan Rajagopalan

**Affiliations:** 1 Department of Immunology, Mayo Clinic, Rochester, Minnesota, United States of America; 2 Department of Dermatology, Mayo Clinic, Rochester, Minnesota, United States of America; 3 Division of Gastroenterology and Hepatology, Mayo Clinic, Rochester, Minnesota, United States of America; 4 Institute for Genomic Biology, University of Illinois, Urbana, Illinois, United States of America; 5 Department of Animal & Range Sciences, College of Agriculture, Montana State University, Bozeman, Montana, United States of America; 6 Division of Endocrinology, Diabetes, Metabolism, & Nutrition, Mayo Clinic, Rochester, Minnesota, United States of America; La Jolla Institute for Allergy and Immunology, United States of America

## Abstract

Human and animal studies strongly suggest that dietary gluten could play a causal role in the etiopathogenesis of type 1 diabetes (T1D). However, the mechanisms have not been elucidated. Recent reports indicate that the intestinal microbiome has a major influence on the incidence of T1D. Since diet is known to shape the composition of the intestinal microbiome, we investigated using non-obese diabetic (NOD) mice whether changes in the intestinal microbiome could be attributed to the pro- and anti-diabetogenic effects of gluten-containing and gluten-free diets, respectively. NOD mice were raised on gluten-containing chows (GCC) or gluten-free chows (GFC). The incidence of diabetes was determined by monitoring blood glucose levels biweekly using a glucometer. Intestinal microbiome composition was analyzed by sequencing 16S rRNA amplicons derived from fecal samples. First of all, GCC-fed NOD mice had the expected high incidence of hyperglycemia whereas NOD mice fed with a GFC had significantly reduced incidence of hyperglycemia. Secondly, when the fecal microbiomes were compared, *Bifidobacterium*, *Tannerella*, and *Barnesiella* species were increased (p = 0.03, 0.02, and 0.02, respectively) in the microbiome of GCC mice, where as *Akkermansia* species was increased (p = 0.02) in the intestinal microbiomes of NOD mice fed GFC. Thirdly, both of the gluten-free chows that were evaluated, either egg white based (EW-GFC) or casein based (C-GFC), significantly reduced the incidence of hyperglycemia. Interestingly, the gut microbiome from EW-GFC mice was similar to C-GFC mice. Finally, adding back gluten to the gluten-free diet reversed its anti-diabetogenic effect, reduced *Akkermansia* species and increased *Bifidobacterium*, *Tannerella*, and *Barnesiella* suggesting that the presence of gluten is directly responsible for the pro-diabetogenic effects of diets and it determines the gut microflora. Our novel study thus suggests that dietary gluten could modulate the incidence of T1D by changing the gut microbiome.

## Introduction

Type 1 diabetes (T1D) is an organ-specific autoimmune disease directed against the pancreatic beta cells that produce the endocrine hormone, insulin. Ultimately, these specialized endocrine cells are destroyed, resulting in hyperglycemia and a life-long dependence upon exogenous insulin [1]. The etiology of T1D is still not determined and is believed to be multifactorial. Nonetheless, among the many factors that are implicated in the etiopathogenesis of T1D, dietary gluten is important for the following reasons. In humans, early exposure to gluten-containing cereals increases the risk of T1D in individuals expressing susceptible HLA alleles [2]. It is also well recognized that there is a strong association between celiac disease, a gluten-sensitive autoimmune disease and T1D, as celiac patients have a 2.4 fold greater chance of developing T1D [3], [4]. A number of studies have shown that celiac patients who were diagnosed with celiac disease later in life (and as a result had a longer exposure to dietary gluten) had a higher rate of T1D than age-matched celiac patients who were diagnosed with celiac disease at a very young age i.e., less than 3 yrs (therefore, these patients were on a gluten-free diet for a much longer period). This would therefore indicate that longer exposures to dietary gluten increase the risk for developing T1D [5]. A strict adherence to a gluten-free diet also results in a significantly lower prevalence of anti-islet antibodies in CD patients. Overall, these human studies strengthen the notion that dietary gluten could be involved in the etiopathogenesis of T1D [6].

Studies on spontaneous animal models of T1D, in both the non-obese diabetic (NOD) strain of mice and in bio-breeding (BB) rats, have also supported an etiological role for dietary gluten in T1D. When maintained on standard chows (which universally contain gluten), these animals have the greatest incidence of diabetes [7], [8], and introduction of a gluten-free diet significantly reduces the incidence of T1D. Based on these human and animal studies it could be concluded that dietary gluten has an etiological role in T1D. However, the mechanisms by which dietary gluten could influence the incidence of T1D are not fully understood.

A flurry of recent studies have demonstrated that the gut microflora plays an important role in shaping of the immune responses as well as in the development of autoimmunity (including T1D) in animal models [9], [10], [11] and humans [12], [13]. Since diet plays a significant role in determining the composition of gut microflora [14], it is possible that dietary gluten could change the composition of gut microflora and thereby contribute to the etiopathogenesis of T1D. Therefore, in the current study, we investigated using NOD mice whether there is an association between dietary gluten, incidence of T1D and the gut microflora. The results strongly support the pro-diabetogenic role of dietary gluten and suggest that dietary gluten could mediate this effect through altering gut microflora.

## Methods

### Mice

Non-obese diabetic (NOD) mice, originally from Jackson Laboratories (Bar Harbor, Maine), were weaned and maintained upon well-defined chows (described below) for at least three generations before introducing them into the current study. All mice were maintained and monitored in a pathogen-free barrier facility. All the experiments were approved by the Mayo Clinic Institutional Animal Care and Use Committee (IACUC). The AAALAC Accreditation Number is 000717, the OLAW Assurance Number is A3291-01 and the study was covered under the Protocol A26508.

### Rodent Diets

One of the gluten-containing chows (GCC) used was a standard mouse chow (Std-GCC) (LabDiet; PMI Nutrition International). The gluten in the chow is derived from wheat middlings, wheat germ, and ground wheat. We used two types of gluten-free chows (GFC), a casein-based GFC (C-GFC), and an egg white-based GFC (EW-GFC). The C-GFC does not contain any gluten, and casein (200 g/kg) is the sole source of protein (AIN-76A, Research Diets, Inc.). The EW-GFC has egg white (200 g/kg) as the protein source and does not contain casein or gluten (Dyets, Inc, Bethlehem, PA). In some experiments, we used a casein-based GFC that was supplemented with gluten (GS-GFC, Dyets, Inc). This GS-GFC had 50 g wheat gluten and 150 g casein per kg chow.

### Measurement of Blood Glucose Levels (BGL)

Glycemic status was monitored biweekly using a glucometer (Bayer, Pittsburgh, PA). Diabetes was diagnosed when two consecutive blood glucose measurements were >250 mg/dL. GraphpadPrism software (Version 5.0a) was used to generate the Kaplan-Meier curves for the incidence of hyperglycemia as well as the associated p value.

### Anti insulin antibodies

Anti-insulin antibody titers was determined by ELISA using recombinant whole human insulin as the target antigen as per standard procedure. Briefly, ELISA plates were coated overnight with recombinant human-insulin (Sigma-Aldrich) at a concentration of 1 μg/ml in 0.1 M Na_2_HPO_4_. Sera were tested at 1∶100 dilution.

### Flow Cytometry

Distribution of CD4+ CD25+ FoxP3+ T lymphocytes in the spleens, mesenteric lymph node cells, and pancreatic lymph node cells were determined by flow cytometry using a kit from eBioscience (San Diego, Ca).

### Analysis of Gastrointestinal Microbiome

Fresh fecal pellets from 20 week-old NOD mice on indicated diets were collected and snap frozen. The composition, richness and diversity of the fecal microbial ecosystem were used as a proxy of the gastrointestinal (GIT) composition and investigated using standard molecular techniques as previously described [15]. Briefly, microbial DNA was extracted using the MoBio UltraClean Soil DNA Kit (MoBio Laboratories Inc., Carlsbad, CA, USA) with a bead-beating step from fecal material collected from each mouse. The V1–V3 regions of the 16S ribosomal RNA gene was amplified from each sample using primers 27F and 534R [16], [17]. The amplicons were sequenced using 454 pyrosequencing technology. The resulting sequences were quality trimmed using the FastX toolkit (available: http://hannonlab.cshl.edu/fastx_toolkit/). Remaining sequences shorter than 200 nucleotides, with homopolymers longer than 6 nucleotides, or containing ambiguous base calls were removed. Sequences were aligned against the silva database (Pruesse) prior to chimera detection or clustering. Potentially chimeric sequences were detected using UCHIME and removed [18]. The remaining reads were pre-clustered as previously described and then clustered using the average algorithm of mother (www.mothur.org) [19]. Operational taxanomic units (OTUs) were defined as sharing >97% sequence average-linkage identity. Bray-Curtis similarity indices were calculated based on OTU composition of each sample. Non-metric multidimensional scaling (nMDS) and analysis of similarities (ANOSIM) based on a Bray-Curtis dissimilarity matrix was used to assess how microbial community compositions differed among subjects and rank the distances calculated using normalized square root transformed community data [20]. Taxonomic classification was inferred using the Ribosomal Database Project (RDP) tool with a bootstrapping cutoff of 0.7. Correspondence analysis of the GIT microbiota was performed using the vegan package in R [21]. Ace and Chao 1 evaluations are indices of richness the species level, and the Shannon diversity index is a mathematical measure of species diversity in the microbial community.

## Results and Discussion

Among the many potential environmental triggers for T1D, diet has been considered a significant contributing factor [7], [22]. Of the various dietary factors, gluten warrants special mention because epidemiological data suggest that early exposure of infants to cereals containing gluten may increase the risk of T1D [23]. Rodent studies have supported this claim [24]. Mechanistically, it remains to be determined how dietary gluten could facilitate the development of T1D. In the current study, we explored whether alterations in the gut microflora could potentially explain the pro-diabetogenic properties of dietary gluten. To our knowledge, the interaction between dietary gluten, gut microbiota and incidence of T1D has not been investigated previously using the Non-obese diabetic (NOD) mice.

**Figure 1:**
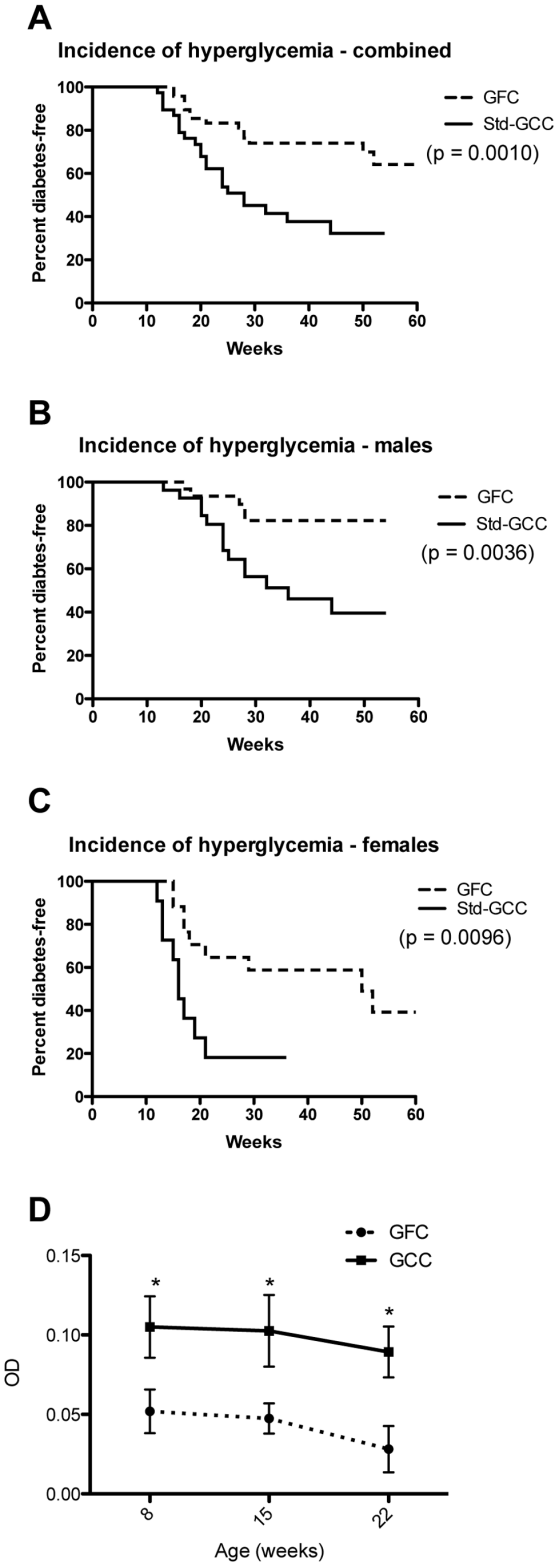
Impact of dietary gluten upon the incidence of hyperglycemia and production of anti-insulin IgG in NOD Mice. Blood glucose levels were measured once every two(gluten-containing) chow (N = 16), and the other that were weaned and maintained upon a gluten-free (casein-based) chow (N = 19). (A) Overall incidence of hyperglycemia. (B) The incidence of hyperglycemia in males and (C) females on the different diets. There were 8 Std-GCC males, 8 Std-GCC females, 8 C-GFC males, and 11 C-GFC females (D). p values are given in parenthesis. Anti-insulin IgG levels were evaluated in the standard (▪, N = 9) and casein-based (▴, N = 8) chow-fed NOD mice every 7 weeks for a total of three time points. P<0.05 for each time point.

First, we established different cohorts of NOD mouse colonies on well-defined diets containing or lacking gluten. As expected, the cumulative incidence of hyperglycemia was higher in NOD mice maintained upon the gluten-containing standard chow, whereas the overall incidence of hyperglycemia was significantly reduced in both male and female NOD mice weaned and maintained upon the casein-based GFC (C-GFC) (Fig 1A–C). As can be seen in Fig 1A–C, the onset of hyperglycemia was also delayed in the NOD mice on the C-GFC. The titers of anti-insulin antibodies, a marker of islet auto-reactivity, were also lower in NOD mice on the C-GFC (Fig 1D).

**Figure 2:**
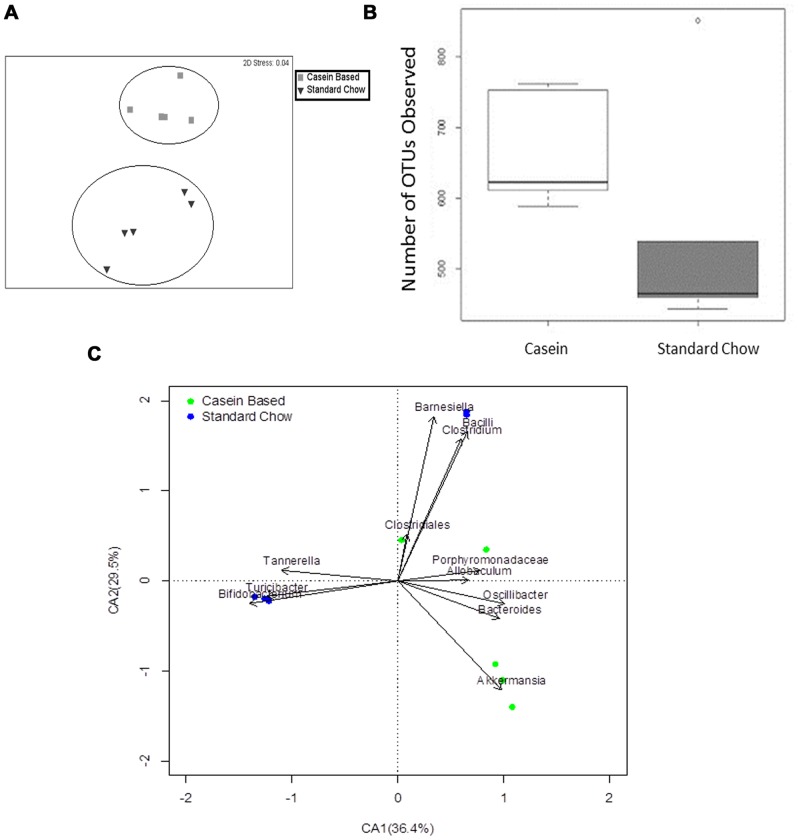
Composition of the intestinal microbiomes in NOD mice on either a casein-based or standard chow. NOD mice were weaned and maintained upon a casein-based gluten-free chow (black squares ▪, N = 5) or a standard gluten-containing chow (black inverted triangle ▾, n = 5). Stool was collected at 20 weeks of age. The microbiomes were evaluated using a multi-dimensional scale analysis as described in methods (Panel A). Richness was determined by observed Operational Taxonomic Units (OTU) (Panel B). Correspondence analysis (CA) plot shows the degree of correlation between specific OTUs and diet (Fig 2C). The black vectors point to the center of gravity of the samples where each OTU mostly occurs. The distance between the tip of the vector and the samples (dots) give an indication of the probability of OTU content in each sample. Green dots represent fecal samples from NOD mice on a casein-based chow; blue dots represent fecal samples from NOD mice on a standard chow. Percentages in parentheses in the CA plot axes describe the amount of variation explained by each axis.

Having confirmed that a gluten-free diet significantly protected mice from spontaneous T1D, we next analyzed the gastrointestinal (GIT) microbiomes from these mice. Bray-Curtis similarity indices clearly showed that the gut microbiomes from C-GFC and standard chow-fed (Std-GCC) mice formed distinct clusters (Fig 2A). The GIT microbiomes of C-GFC mice were more similar to each other than to those of the Std-GCC mice (Analysis of Similarities (ANOSIM) Global R: 0.852, P = 0.001). Also, the C-GFC microbiomes had greater observed and estimated total richness as determined by the number of observed Operational Taxonomic Units (OTUs) (Fig 2B), ACE and Chao1 richness evaluations (Fig S1A–S1B in File S1). However, there was no difference in the diversity as determined by the Shannon Diversity Index (Fig S1D in File S1). There was also no difference in the number of sequences obtained for the two dietary groups (Fig S1E in File S1). Most importantly, a correspondence analysis (CA) plot of genus-level microbial diversity demonstrated that each dietary group was associated with specific genera (Fig 2C). Increases in *Akkermansia* were observed in C-GFC NOD mice, whereas *Barnesiella, Bifidobacterium, Tannerella* and *Turcibacter* were seen in (Std-GCC) (Fig 2C). Taken together, these data suggested that the microbiomes of NOD mice fed the diabetes-inhibitory C-GFC diet were richer and distinct from mice fed a pro-diabetogenic gluten-containing standard chow.

**Figure 3:**
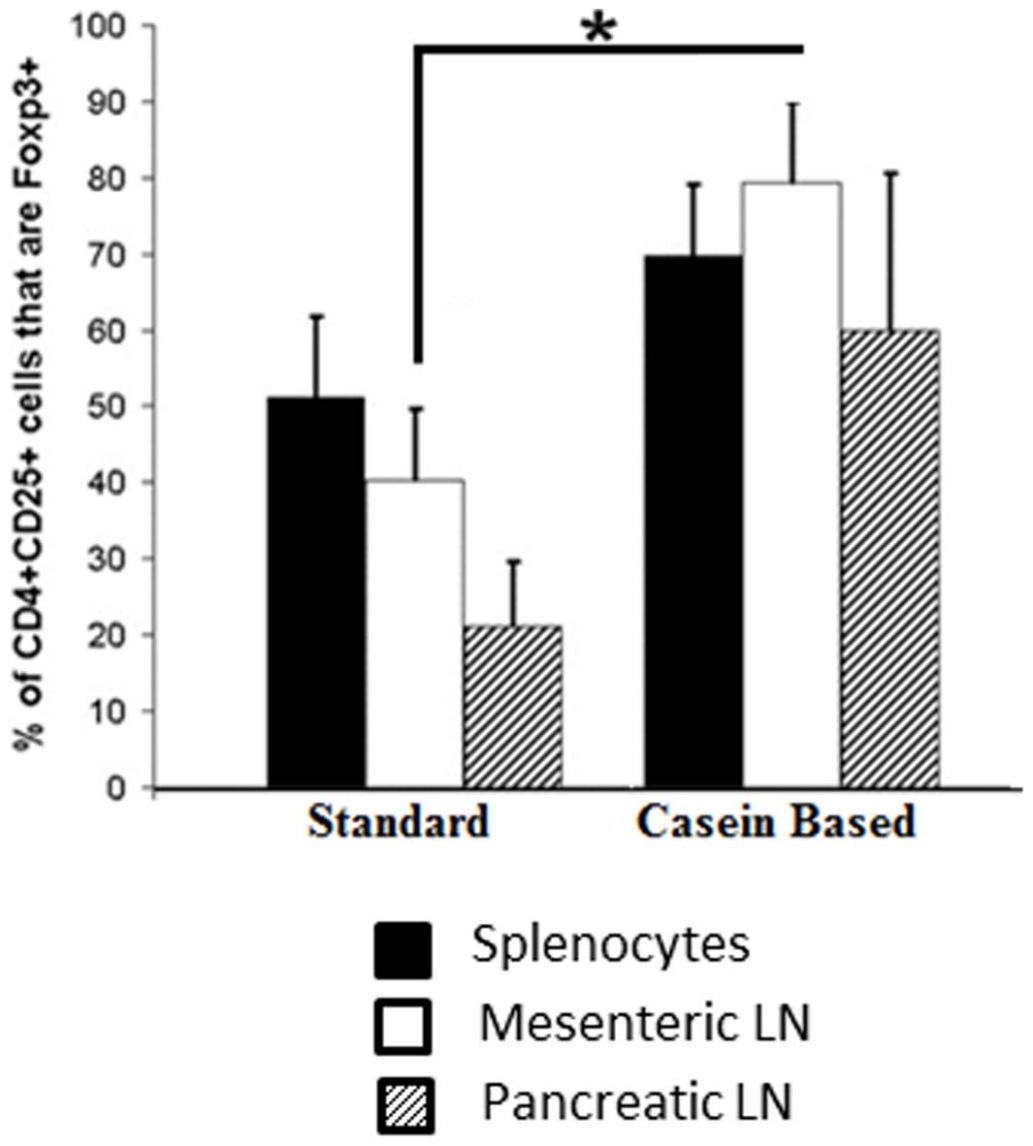
Regulatory T lymphocytes and diet. The distribution of regulatory (CD4^+^ CD25^+^ Foxp3^+^) was compared by FACS analysis between NOD mice on a casein-based or a standard chow. * p<0.05.

Studies done using germ-free mice and specific pathogen-free mice have identified a very important role for gut microbiota in shaping of the T cell repertoire as well as in the development and homeostasis of immunoregulatory T cells (Tregs) [9]. In our study, NOD mice weaned and maintained on C-GFC had a significantly higher percentage of CD4^+^ CD25^+^ Foxp3^+^ cells in the mesenteric lymph nodes, which drain the gut, when compared to the standard chow fed NOD mice (Fig 3, p = 0.02). CD4^+^ CD25^+^ Foxp3^+^ cells were also increased in the spleen and pancreatic lymph nodes of C-GFC mice when compared to standard chow mice, but these differences were borderline significant (p = 0.06 and 0.09 respectively). Thus, there was a strong association between dietary gluten, incidence of diabetes, microbiome composition and the numbers of T regulatory cells.

**Figure 4:**
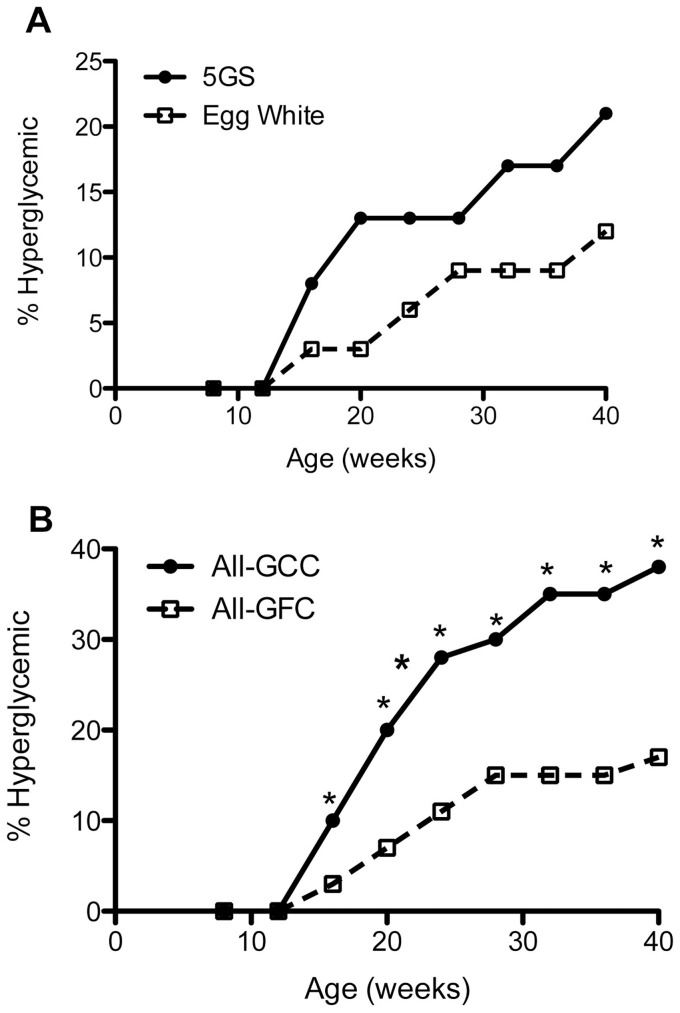
Reversal of the anti-diabetogenic effects of gluten-free chow by supplementation with gluten and overall effective of gluten-free chow on the incidence of hyperglycemia in NOD mice. (A) Blood glucose levels were measured every two weeks for two cohorts of mice: mice that were weaned and maintained upon an egg white-based gluten-free (EW-GFC) chow (N = 19) and a casein-based gluten-free chow supplemented with gluten (GS-GFC) (N = 23). The overall incidence of hyperglycemia is depicted. (B) Diabetes incidence data from all diet groups were pooled and presented as gluten-free (both GFC-C and EW-GFC) (N = 51) and gluten-containing (standard GCC and GS-GFC) (N = 39) P<0.05 for each time point.

It could be argued that it is not the absence of gluten, but the presence of casein in the C-GFC diet determined the microbiome that associated with low incidence of T1D. To address this issue, we used two small cohorts of NOD mice, one raised on an egg white-based GFC (EW-GFC) and the other raised on a casein-based GFC that was supplemented with gluten (GFC-GS). The incidence of hyperglycemia was studied. As can be seen from Fig 4A, the overall incidence of hyperglycemia was still reduced in EW-GFC. Moreover, supplementation of gluten abolished the protective effect of C-GFC. The cumulative incidence of hyperglycemia was significantly reduced in female NOD mice on EW-GFC compared to GS-GFC-fed NOD mice. Only 3/10 (30%) females developed hyperglycemia in EW-GFC group, compared to 5/7 (71.43%) in GFC-GS group (p<0.05) at 40 weeks of age. However, there was no difference in the diabetes incidence in males; 3/20 (15%) males developed hyperglycemia in EW-GFC group, compared to 2/15 males (13.3%) in the GFC-GS fed mice (p  =  NS). When the data from all diet groups were pooled, we could clearly see a diabetes-protective trend with gluten-free diets (C-GFC and EW-GFC) compared to the gluten-containing diets (Std-GCC and GFC-GS) (Fig 4B). As both casein-based GFC as well as egg white-based GFC had low incidence of diabetes and supplementing gluten to the casein-based GFC reverted this effect, we could conclude that it is the absence of gluten and not the presence of casein in the diet that associates with low incidence of hyperglycemia.

**Figure 5:**
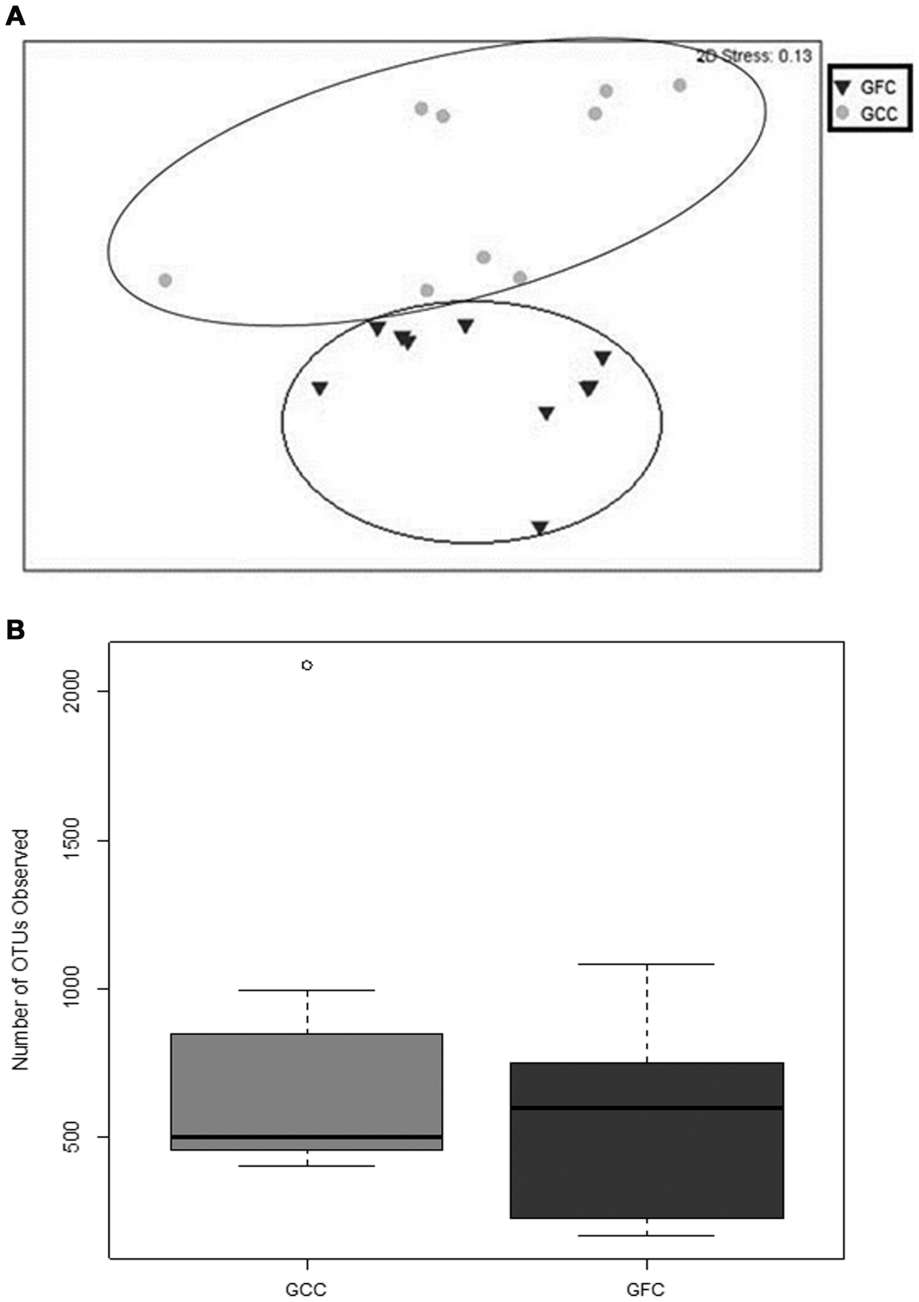
Effect of dietary gluten upon the composition of the intestinal microbiomes in NOD mice. Microbiomes of mice on gluten free chows were compared with those of mice on gluten containing chows. For the gluten free chow derived microbiomes, microbiomes from five mice on an egg-white based gluten-free chow and five from a casein-based gluten-free chow were grouped together to make a total of 10 gluten-free chow mice (▾). For the microbiomes derived from mice on gluten containing chows, five were derived from mice on standard (gluten containing) chow and five were derived from mice on gluten-supplemented casein-based chow, making a total of 10 gluten associated microbiomes (•). These were evaluated using a multi-dimensional scale analysis (Fig 5A). Richness was determined by observed Operational Taxonomic Units (OTU) (Fig 5B).

**Figure 6:**
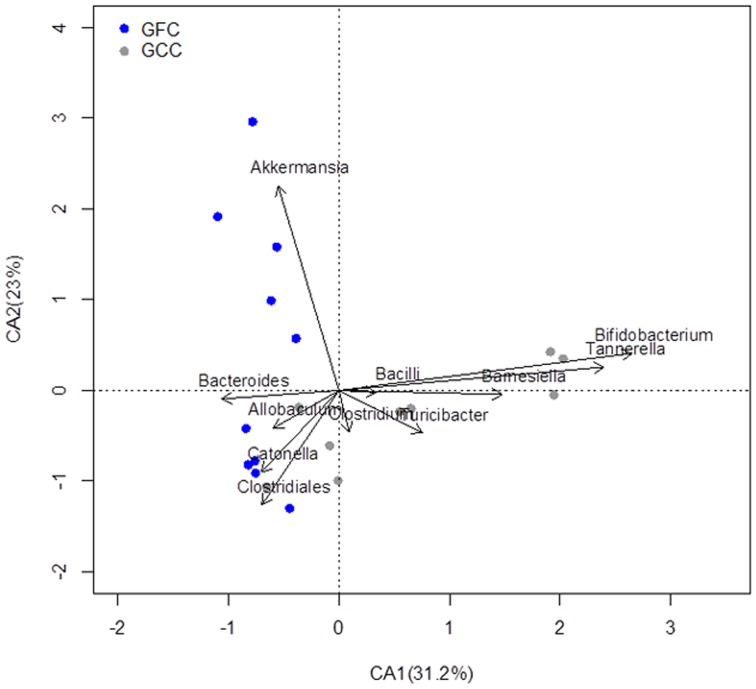
Effect of Dietary Gluten Upon Specific OTU (Genus) Abundance. A. Correspondence analysis (CA) plot shows the degree of correlation between specific OTUs and diet in all of the gluten-containing chows group (GCC) and gluten-free chows group (GFC) as in fig 5.

**Figure 7:**
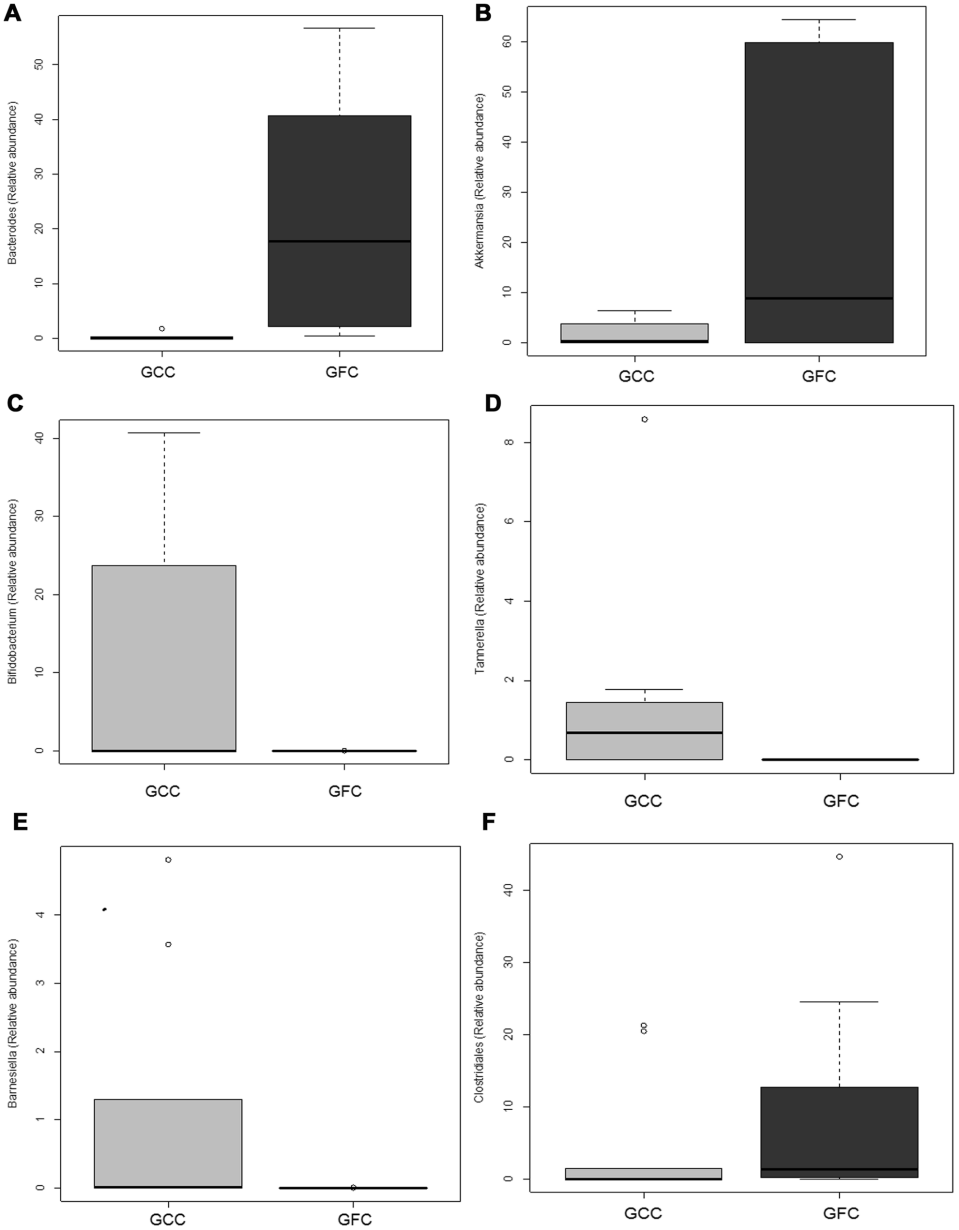
Effect of Dietary Gluten Upon Specific OTU (Genus) Abundance. Mean relative abundance for each of 6 genera in all of the gluten-containing chows group (GCC) and gluten-free chows group (GFC) as in fig 5 are plotted in a box and whisker plot. The six genera plotted are A) Bacteroides, B) Akkermansia, C) Bifidobacterium, D) Tannerella, E) Barnesiella, F) Clostridiales. The respective p values are: A) 0.005843 B) 0.01783 C) 0.03615 D) 0.01761 E) 0.0234 and F) 0.0541.

We next analyzed the microbiomes of (EW-GFC) and the (GFC-GS) mice. Bray Curtis similarity analysis of the GIT microbiomes from these four different dietary groups demonstrated that each dietary group clustered together with few outliers (Fig S2 in File S1). We next pooled the microbiome data from all experimental groups and investigated the effect of gluten (either presence or absence) on the microbiomes. Interestingly, the microbiomes from (EW-GFC) mice clustered together with C-GFC (both diabetes-protective diets), whilst the microbiomes of the NOD mice fed GFC-GS clustered with those of the Std-GCC fed mice (both diabetogenic diets) when analyzed by Bray-Curtis method (ANOSIM Global R: 0.383, P = 0.001 Fig. 5A). However, there was no observed difference in the richness of the two groups when using the number of OTUs (Fig. 5B) or by ACE and Chao1 Richness analyses (Fig S3A–S3B in File S1). Shannon diversity analysis also did not show a difference in the diversity between the GFC and GCC groups of GIT microbiomes (Fig. S3D in File S1). Total number of sequences obtained also did not differ between the two groups (Fig. S3E in File S1). Correspondence analysis (CA) of the Genus-level GIT microbiomes also revealed that *Bacteroides* and *Akkermansia* significantly corresponded with a gluten-free diet (p = 0.01 and 0.02 respectively, Welch two sample t Test), while *Bifidobacterium* (p = 0.04), *Tannerella* (p = 0.02), and *Barnesiella* (p = 0.02) corresponded with GCC (Fig. 6 and 7A–F). *Clostridiales* also trended toward GCC, but was not significant (p = 0.0541). In summary, the microbiomes in mice receiving any type of diet were diverse. The microbiomes in mice fed gluten-free diets (casein-based or egg white-based, both of which reduce the incidence of T1D), clustered together and comprised predominantly of *Akkermansia*. However, the microbiomes from all gluten-containing diets (Std-GCC or GFC-GS), both of which associated with a high incidence of T1D, formed independent clusters comprising of different genera, predominantly *Bifidobacterium*, *Tannerella* and *Barnesiella*.

Recent microbiome studies on NOD mice have shown that certain bacteria are associated with protection from diabetes, as opposed to a “pathogenic” association (i.e., associated with the diabetic phenotype) [10], [13]. Particularly, Hansen et al [25] recently demonstrated that an early treatment with vancomycin significantly reduces the incidence of T1D in NOD mice and such protected mice had increased the level of *Akkermansia muciniphilia* in their gut microbiome. Based on this observation and our findings, we could propose that gluten could contribute to the pathogenesis of T1D in the NOD mouse by decreasing *Akkermansia*, a genus of GIT microbiota that protects against T1D. Alternatively, gluten-containing diets may promote “pathogenic or diabetogenic” bacteria. Further experiments are needed to prove these possibilities. In conclusion, we have shown that gluten-free diets significantly delay the onset as well as reduce the overall incidence of spontaneous T1D in NOD mice; the frequency of T regulatory cells was increased in such protected mice, supporting another paper that T regulatory cells inhibit the development of insulitis and T1D [26]. Gut microbiomes from mice fed gluten-free diets was distinct from those of mice fed diabetogenic, gluten-containing diets. Therefore, gluten could contribute to the pathogenesis of T1D by modulating the gut microflora.

## Supporting Information

File S1 *Figure S1, Additional Analyses of the Richness and Diversity of the Intestinal Microbiomes of Mice on Casein Based or Standard Chows.* Further analyses of the richness of the two dietary groups of microbiomes were conducted: ACE (Fig. S1A), Chao1 (Fig. S1B), and additional OTUs (Fig. S1C). Alpha diversity was determined using the Shannon diversity index (S1D). Total number of sequences obtained is plotted in figure S1E. *Figure S2, Bray Curtis Similarity Analyses of the gluten supplemented and egg-white based chows.* Further analyses were done on the similarity of the gluten supplemented with the casein based gluten-free chow (Fig. S2A), the similarity of the egg white based gluten-free chow with the casein based gluten-free chow (Fig. S2B), the similarity of the gluten supplemented chow with the standard chow (Fig S2C), and the similarity of the standard chow with the egg white based chow (Fig S2D). *Figure S3, Additional Analyses of the Richness and Diversity of the Intestinal Microbiomes of Mice on GCC and GFC.* More evaluations of richness were conducted: ACE (Fig. S3A), Chao1 (S3B), and Additional OTUs (S3C). Alpha diversity was determined using the Shannon diversity index (S3D). Total number of sequences obtained is plotted in figure S2E.
(PDF)
