# Supplementary material for: Low Incidence of Spontaneous Type 1 Diabetes in Non-Obese Diabetic Mice Raised on Gluten-Free Diets Is Associated with Changes in the Intestinal Microbiome
Source: PLoS One. 2013 Nov 13;8(11):e78687. doi: 10.1371/journal.pone.0078687 (PMC3827256; doi:10.1371/journal.pone.0078687)
Supplement: File S1 — Figure S1, Additional Analyses of the Richness and Diversity of the Intestinal Microbiomes of Mice on Casein Based or Standard Chows. Further analyses of the richness of the two dietary groups of microbiomes were conducted: ACE (Fig. S1A), Chao1 (Fig. S1B), and additional OTUs (Fig. S1C). Alpha diversity was determined using the Shannon diversity index (S1D). Total number of sequences obtained is plotted in figure S1E. Figure S2, Bray Curtis Similarity Analyses of the gluten supplemented and egg-white based chows. Further analyses were done on the similarity of the gluten supplemented with the casein based gluten-free chow (Fig. S2A), the similarity of the egg white based gluten-free chow with the casein based gluten-free chow (Fig. S2B), the similarity of the gluten supplemented chow with the standard chow (Fig S2C), and the similarity of the standard chow with the egg white based chow (Fig S2D). Figure S3, Additional Analyses of the Richness and Diversity of the Intestinal Microbiomes of Mice on GCC and GFC. More evaluations of richness were conducted: ACE (Fig. S3A), Chao1 (S3B), and Additional OTUs (S3C). Alpha diversity was determined using the Shannon diversity index (S3D). Total number of sequences obtained is plotted in figure S2E. (PDF) [file pone.0078687.s001.pdf]

Supplementary Figures:

Fig. S1A:

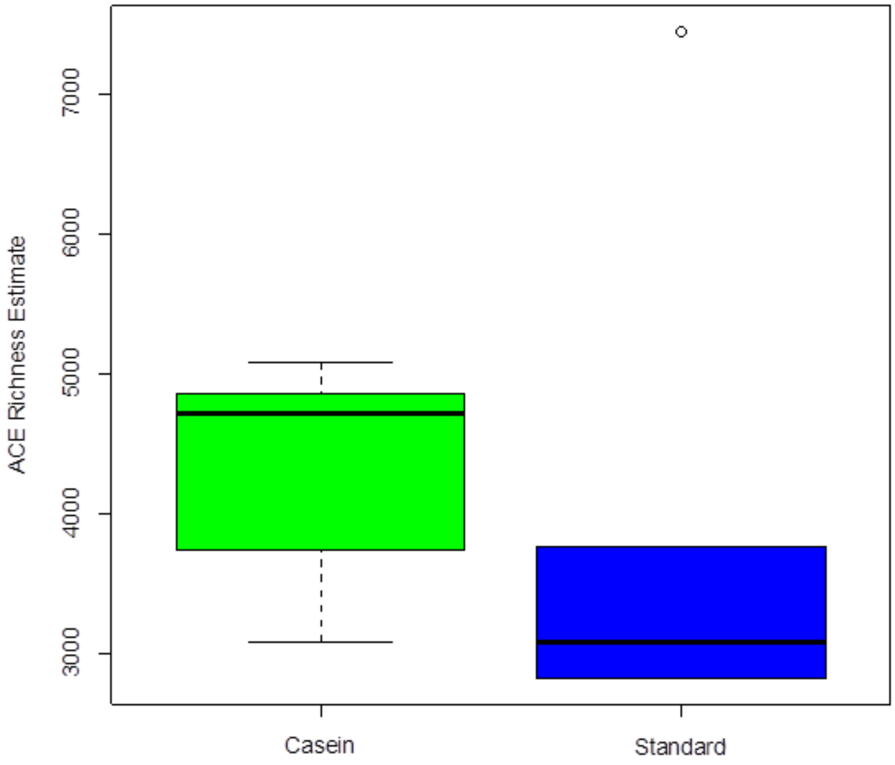

Fig. S1B:

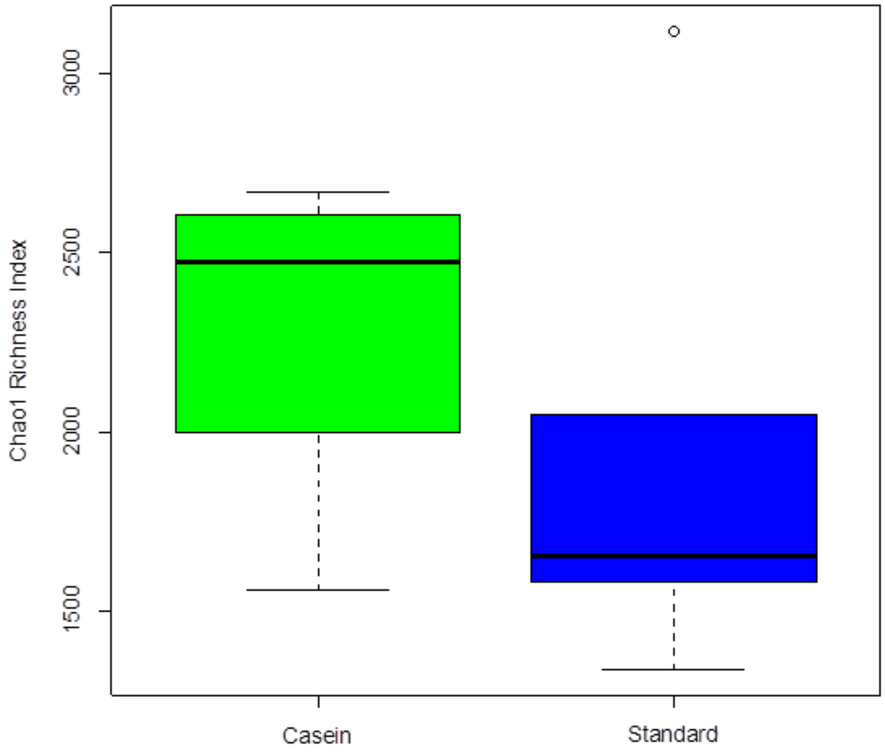

Fig. S1C:

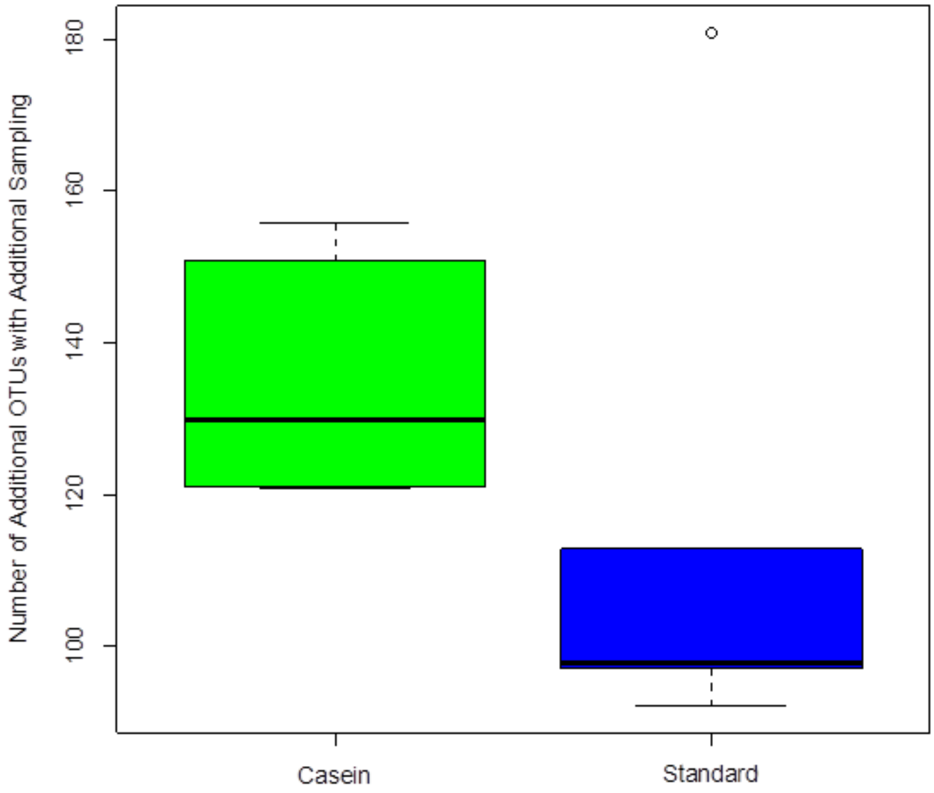

Fig. S1D:

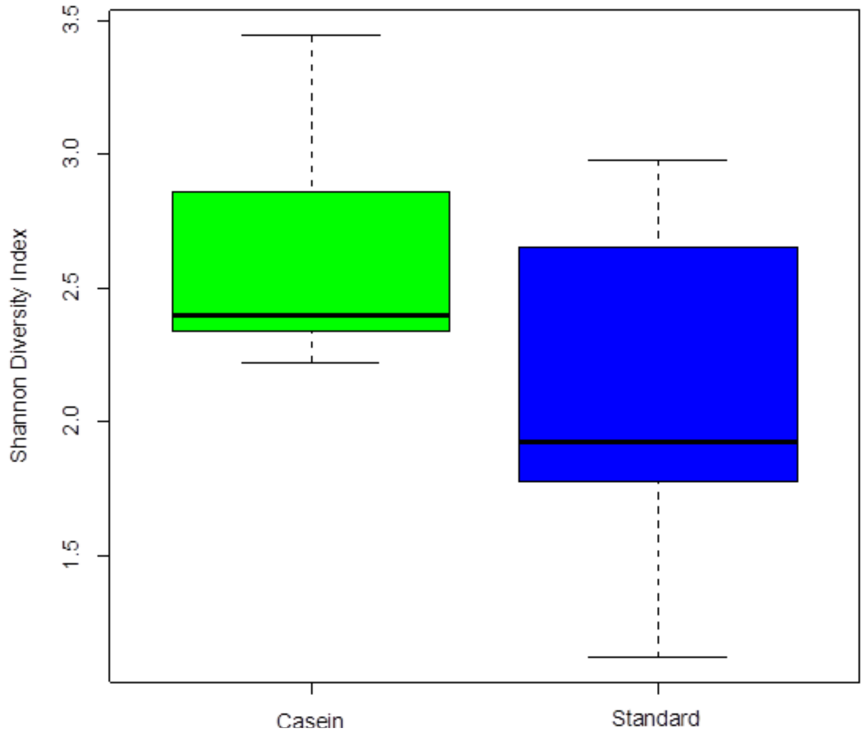

Fig. S1E:

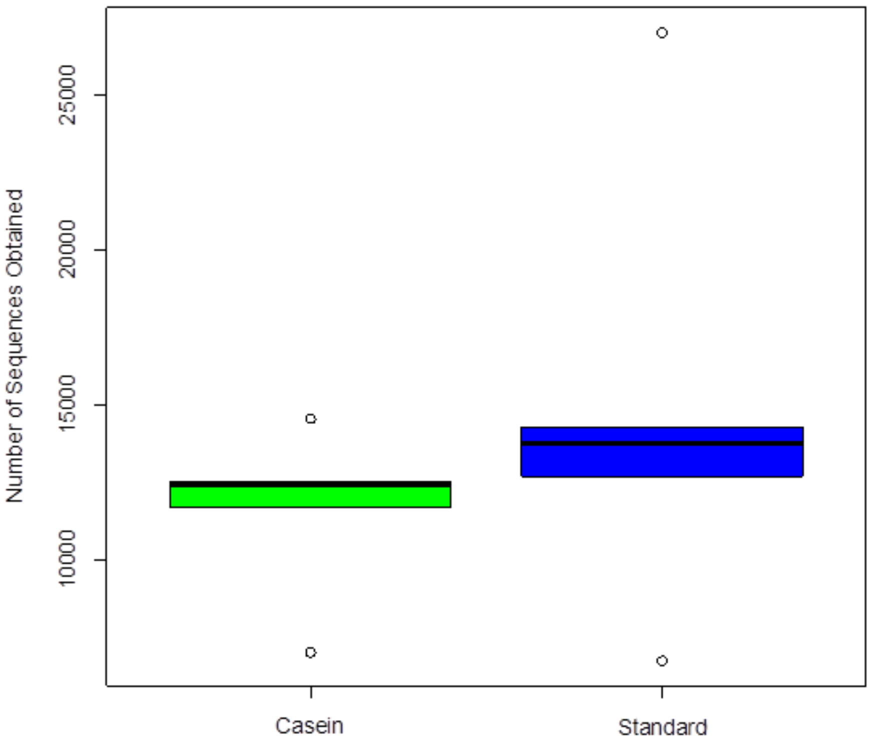
25000
20000
15000
10000
Number of Sequences Obtained
Casein
Standard

Fig. S2A:

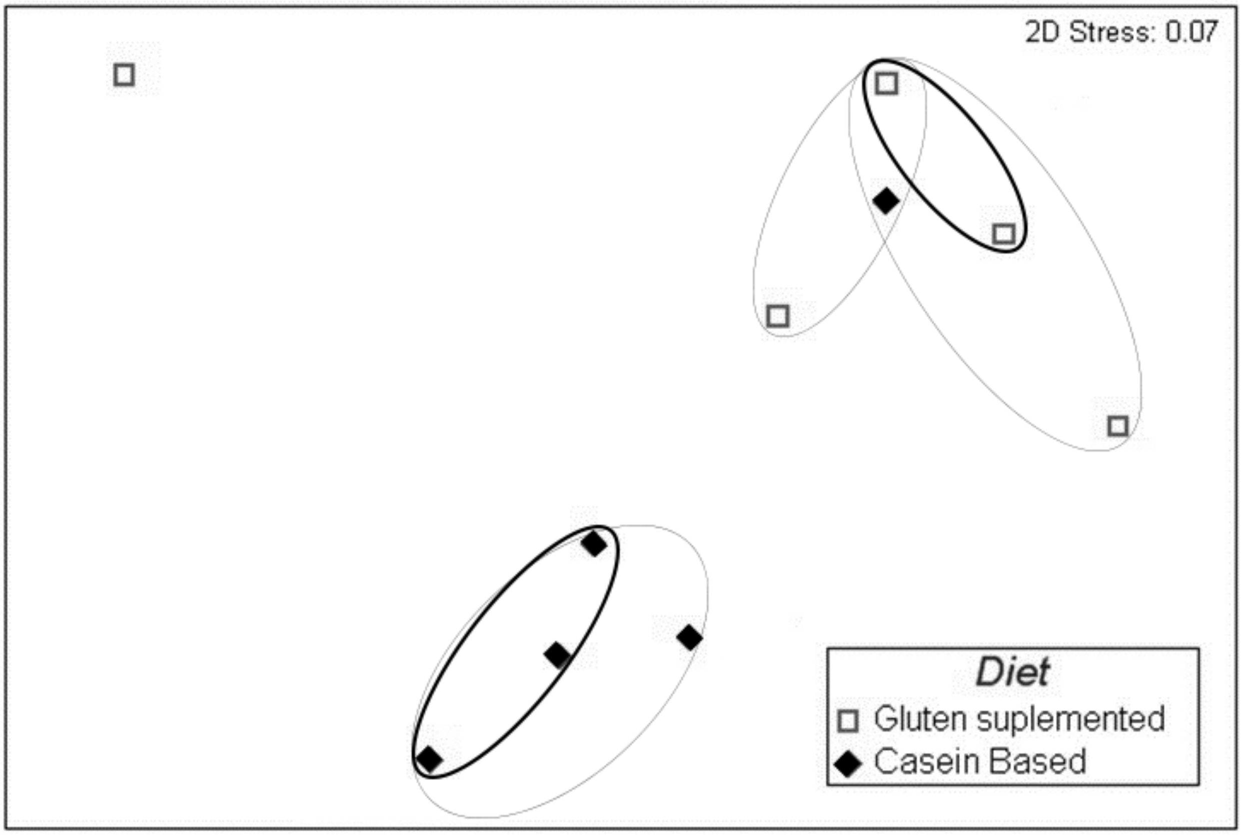

Fig. S2B:

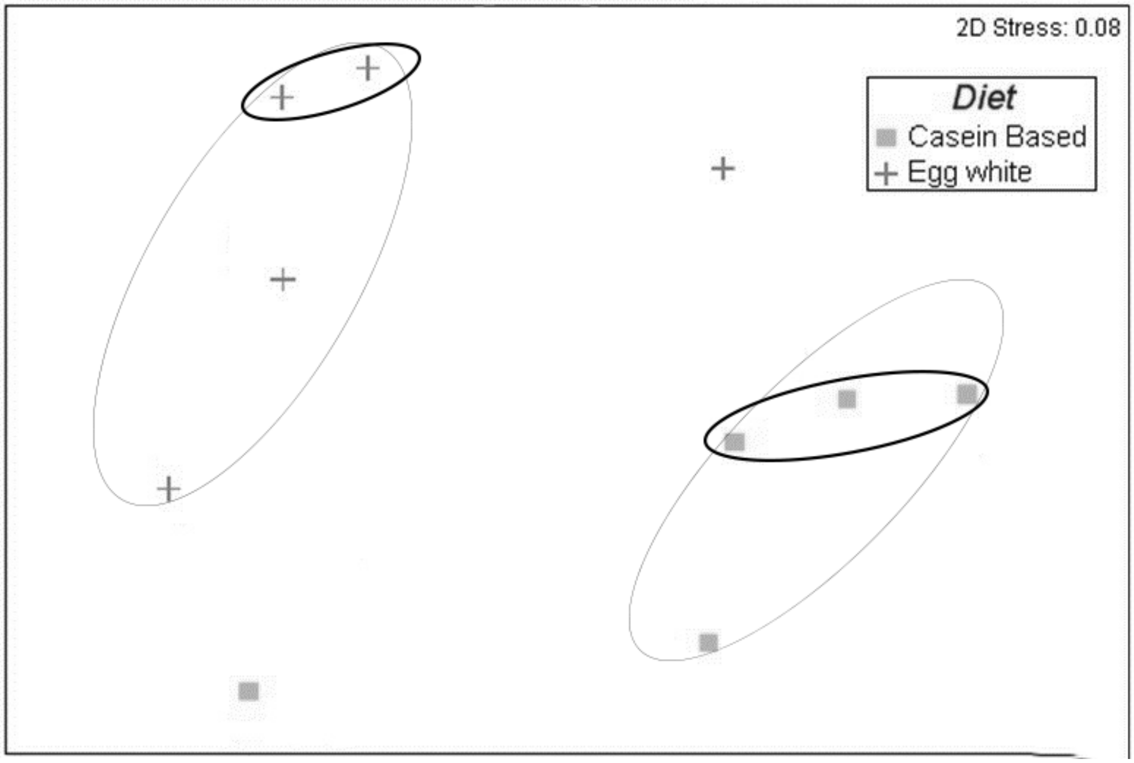

Fig. S2C:

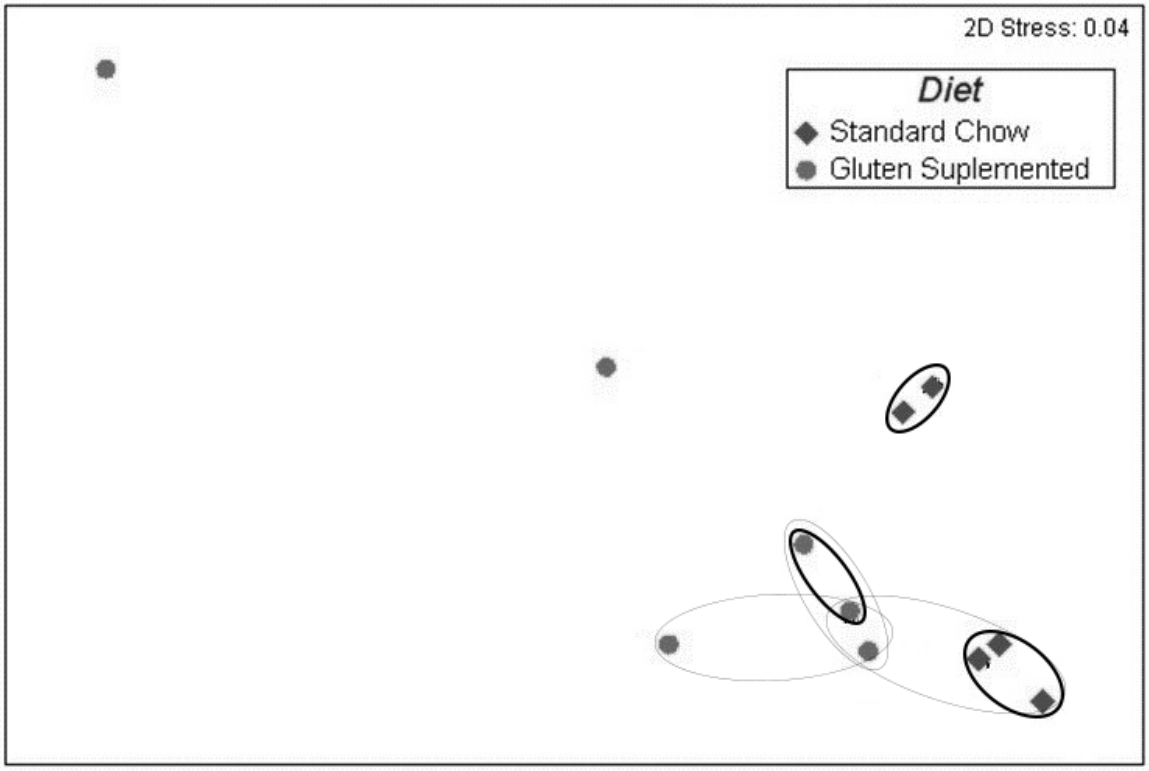

Fig. S2D:

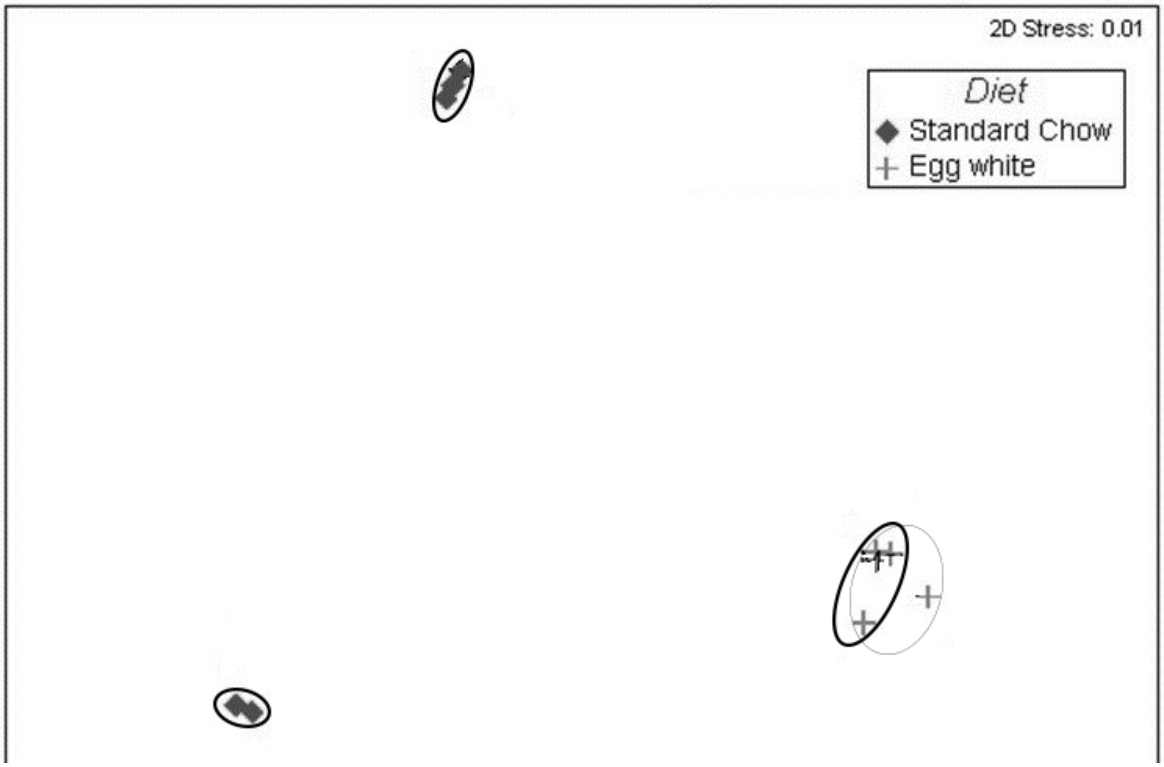

Fig. S3A:

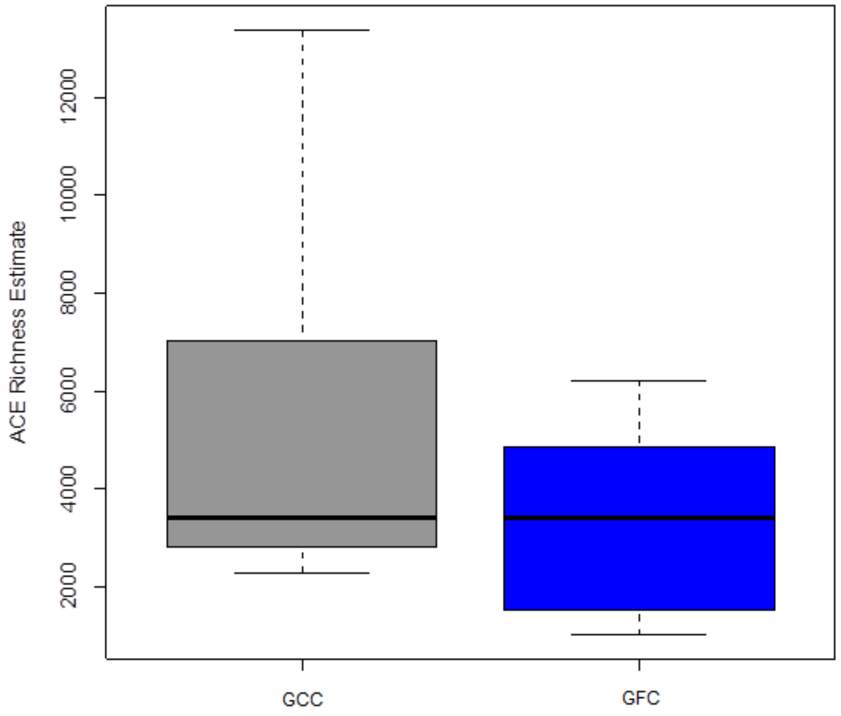

Fig. S3B:

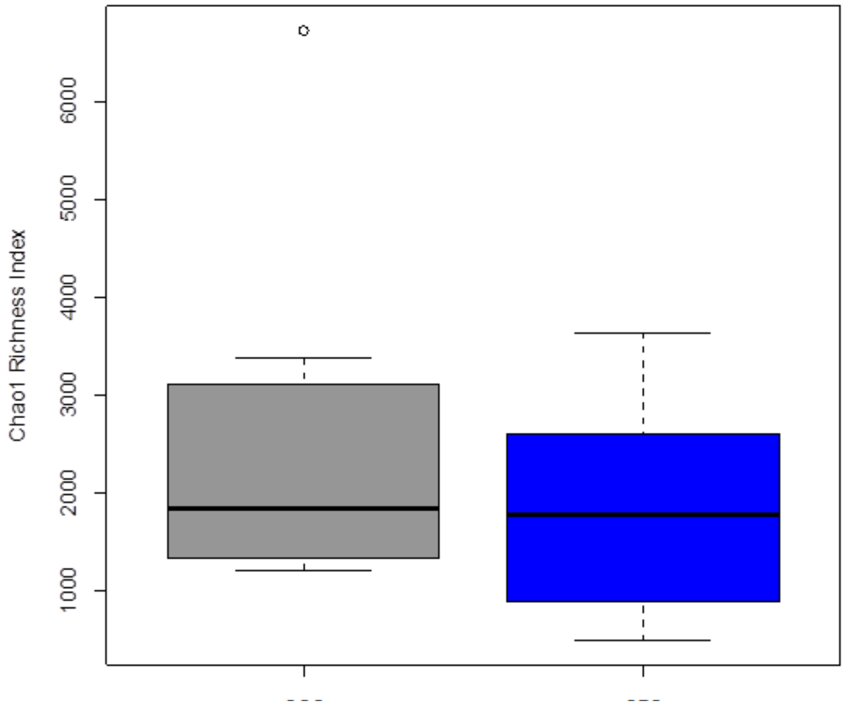

Fig. S3C:

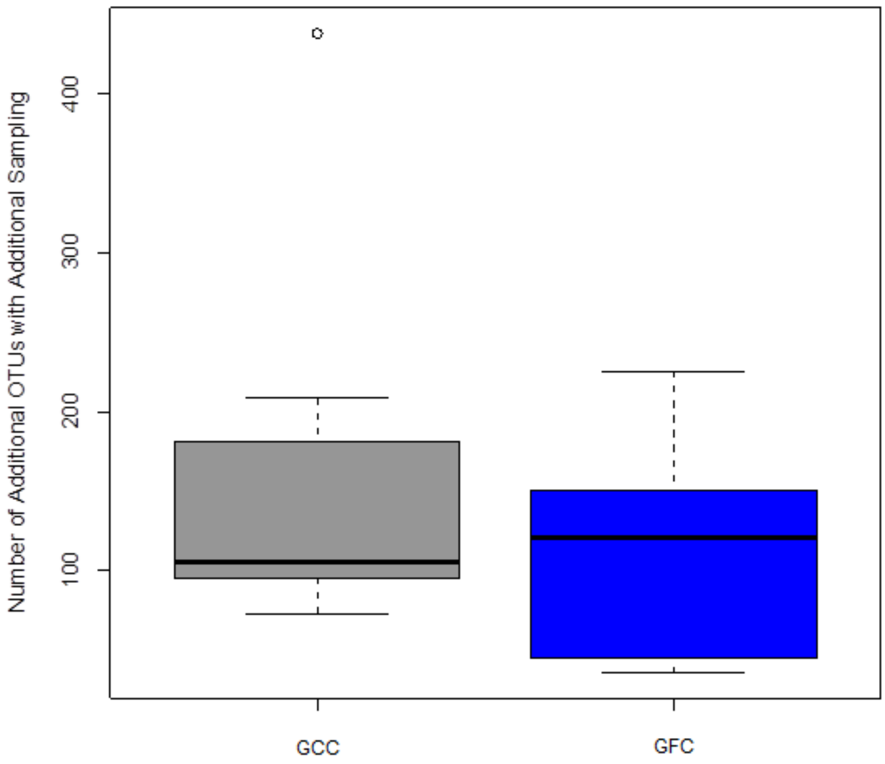

Fig. S3D:

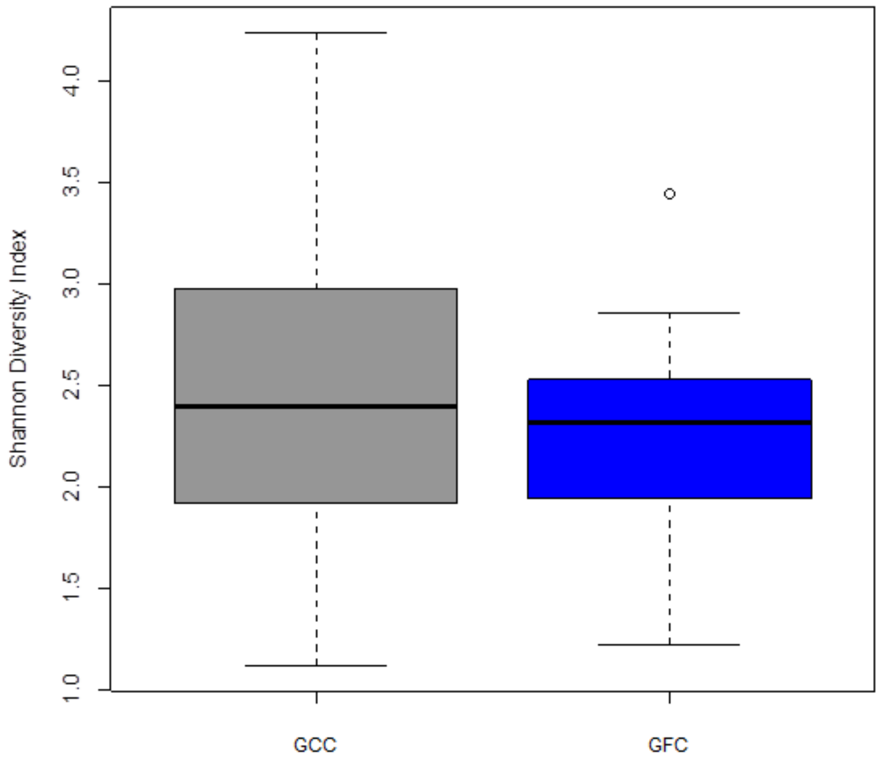

Fig. S3E:

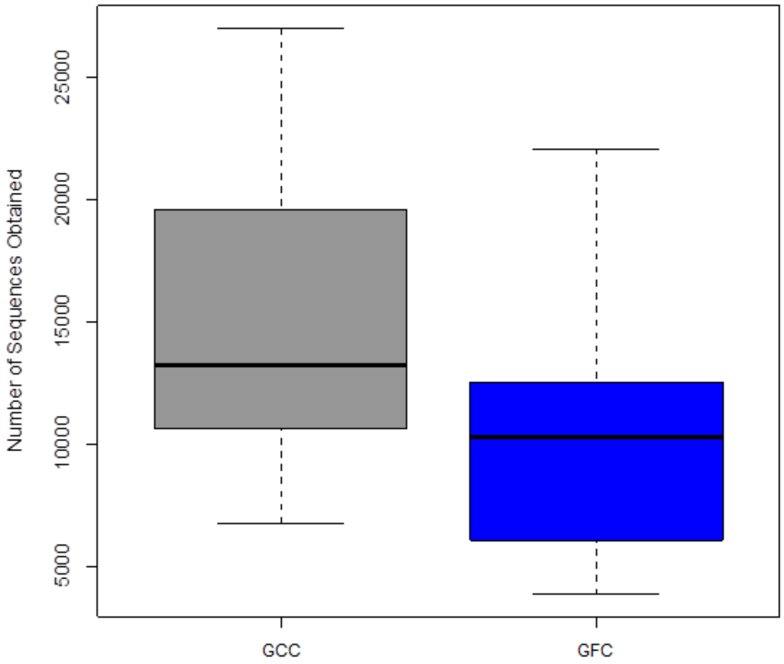
Number of Sequences Obtained
25000
20000
15000
10000
5000
GCC
GFC
